# The Transporter Classification Database

**DOI:** 10.1093/nar/gkt1097

**Published:** 2013-11-12

**Authors:** Milton H. Saier, Vamsee S. Reddy, Dorjee G. Tamang, Åke Västermark

**Affiliations:** Department of Molecular Biology, University of California at San Diego, La Jolla, CA 92093-0116, USA

## Abstract

The Transporter Classification Database (TCDB; http://www.tcdb.org) serves as a common reference point for transport protein research. The database contains more than 10 000 non-redundant proteins that represent all currently recognized families of transmembrane molecular transport systems. Proteins in TCDB are organized in a five level hierarchical system, where the first two levels are the class and subclass, the second two are the family and subfamily, and the last one is the transport system. Superfamilies that contain multiple families are included as hyperlinks to the five tier TC hierarchy. TCDB includes proteins from all types of living organisms and is the only transporter classification system that is both universal and recognized by the International Union of Biochemistry and Molecular Biology. It has been expanded by manual curation, contains extensive text descriptions providing structural, functional, mechanistic and evolutionary information, is supported by unique software and is interconnected to many other relevant databases. TCDB is of increasing usefulness to the international scientific community and can serve as a model for the expansion of database technologies. This manuscript describes an update of the database descriptions previously featured in NAR database issues.

## INTRODUCTION: THE TC SYSTEM: DESIGN AND RATIONALIZATION

In 1995, Fleischmann *et al.* (1) published the full genome sequence of a living organism, *Haemophilus influenzae*, the first time such a feat had been accomplished. This revolutionary event marked the beginning of the genomics era. Because of our long-standing interest in molecular transmembrane transport, members of the Saier laboratory recognized the need for a classification system for transport systems equivalent to the Enzyme Commission (EC) system already in existence for enzymes (2). The EC system classified enzymes strictly on the basis of function, as it was designed before sequence and phylogenetic data were available. Even before the advent of the genomics revolution, it became clear that the EC system was tremendously deficient because it could not accommodate phylogenetic data without restructuring the entire system. Although considered desirable by many, such a restructuring of the EC system has never been achieved.

Even before 1995, our laboratory was conducting phylogenetic analyses of transport proteins [for review, see (3)]. We realized that phylogeny reflects protein structure, function and mechanism, and therefore, is an essential component of any molecular classification system. With a desire to conduct whole genome analyses of transporters, we recognized a need for a universal system of transport protein classification that took cognizance of both function and phylogeny. With this conviction in mind, we designed what is now known as the Transporter Classification (TC) system.

Transporters in the TC Database (TCDB) are classified using a functional/phylogenetic five-tier system (4,5) as follows: N1.L1.N2.N3.N4, where N is a number and L is a letter: N1 is the class; L1 is the subclass; N2 is the family (sometimes actually a superfamily); N3 is the subfamily; (or family in the case of a superfamily) and N4 is the actual transport system. Classes 1–5 are well defined (channels, secondary carriers, primary active transporters, group translocators and transmembrane electron carriers, respectively); classes 6–7 are presently empty, being reserved for yet to be discovered classes, and classes 8 and 9 represent accessory proteins and incompletely characterized proteins, respectively. This system, describing transport systems from all types of living organisms, was formally adopted by the International Union of Biochemistry and Molecular Biology (IUBMB) in June 2001 and has served the international scientific community effectively ever since (6–9).

## DATABASE CONTENT AND ACCESS

**Figure 1.**
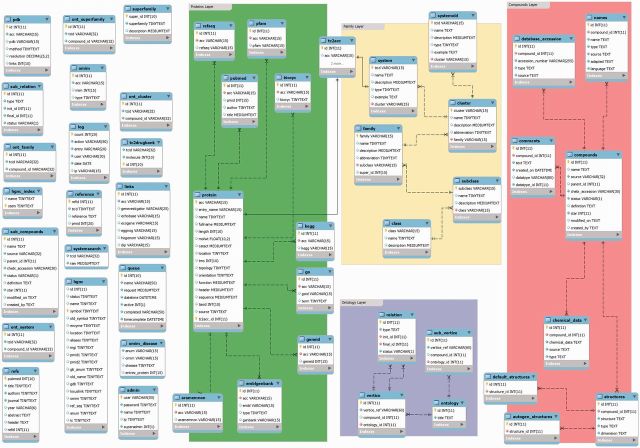
Current MySQL schema, displayed using Workbench 6.0 CE and showing the tables currently in TCDB’s database architecture. Each line in a table represents a column and displays which datatype (such as int, varchar, text, etc.) can be stored. Ten tables, which are not being used directly by TCDB but that have been used for maintenance tasks are not shown in the diagram: test, lang error, proteinold, tc2acc broke, tc2acc 1, flags, cflags, temp_tms, temp_preds and misc. A table that has a trifork (entity relationships) pointing toward it contains a column with explicit IDs from another table. The tables having no entity relationships are grouped on the left. The diagram contains four layers (left to right, and from top to bottom): the protein layer (green), the family layer (yellow), the ontology layer (blue) and the compounds layer (red).

Encoded within the relational database schema is the functional/phylogenetic TC taxonomy (Figure 1). Users can access the information through our intuitive interface, where information can be viewed at different levels of granularity by returning populated HTML data to the web browser client (the superficial tier). Users can enter at the top levels for information about classes and families and descend to the deepest level about individual proteins.

Since its last publication in the NAR database issue in 2009 (5), there has been significant change in the database design (schema above). Some basic issues pertaining to data integrity, redundancy and management have led to conversion of the MySQL Table Engine from MyISAM to InnoDB. Perhaps the most important justification for this conversion is the fact that different levels of TC classification have a type of parent–child relationship. A foreign key constraint should allow cascading action when a row (tuple) is inserted/updated/deleted. Thus, all related tables are affected, leaving no orphaned records. Roughly one half of the schema follows the standard relationships between class, subclass, superfamily, family, cluster or subfamily and system, and the other half shows tables of information pertaining to unique UniProt protein accession numbers.

The steps involved and basic ideas behind the TCDB Admin interface for curation are the same as above and follow the DB design schema. However, the look and feel of the interface has changed since its update in 2010, along with some new options such as ‘View Task Queue’ and ‘View Staff Logs’. We share our mapping file with different databases, and these files are automatically updated every time a new protein is added to the database.

The entire web interface has been revamped. The new look and feel should be consistent across all major browsers, easier to navigate, URL friendly, and overall, a huge improvement from the previous HTML frame-based web pages. For example, the browse tab for viewing the TC System (http://www.tcdb.org/browse.php) has been entirely redesigned using jQuery. For a more detailed description of the capabilities available to the user, see Wakabayashi *et al.* (10).

In addition to the search option under the search tab, one can search TCDB from a search box on the main page using single or multiple terms including TC ID#, key word, protein name or abbreviation, organismal source, author name, UniProt accession number, PDB ID number, associated disease, reference, etc. The following details are returned with a protein search, or can be easily accessed following such a search: 

(i) TC ID#, (ii) reference, (iii) accession number, (iv) protein name, (v) length, (vi) molecular weight, (vii) species, (viii) predicted number of TMSs, (ix) location/topology/orientation and (x) database of interacting proteins (DIPs) and Pfam reference.

The user is also given an option of either BLASTING/PSI-BLASTING the protein against the non-redundant National Center for Biotechnology Information (NCBI) or TCDB (accessed from the sidebar). Additional analysis options, such as predicting number of TMSs through hydropathy plots, are also available (see below).

TCDB collaborates with many important databases (see Reference #10 for recent technical improvements), and shares cross-database links with them; these are available on the individual protein pages. Internal hyperlinks connecting references to classes, families and proteins have been updated.

## RECENT TECHNICAL IMPROVEMENTS (2011–13)

We have:

1. Incorporated an improved administration page, built-in semi-automatic machine learning tools (11) and user contributions, allowing protein history tracking, see Wakabayashi *et al.* (10).
2. Updated software to BLAST 2.2.27.
3. Replaced the WHAT program (12) with a functionally similar python version to increase speed and reliability.
4. Made the TCDB BLAST database available, generated in real-time.
5. Made the TMSTATS Program (13) available for analyzing topological (TMS) statistics using three different topological prediction programs, HMMTOP (14), MEMSAT (15) and SPOCTUPUS (16), giving histograms of TMS distribution for any protein or for any TC class, subclass, family, subfamily or any combination of these.
6. Made Global Sequence Alignment Tool (GSAT) (13) available for performing pairwise alignments. GSAT performs a shuffle-based alignment to detect distant homologs using the Needleman and Wunsch algorithm.
7. Implemented Protocols 1/2: Protocol 1 runs a PSI-BLAST search of the NCBI protein database with iterations, collects results, removes redundant/small/similar sequences, annotates, tabulates and counts TMSs. Protocol 2 allows the rapid identification and quantitative evaluation of homologs between any two FASTA files using the GSAT program (13).
8. Established a homology section that replaces the GAP (17) and ICC programs with GSAT and Protocol 2 (13), and included class-wide comparisons that can be performed with these programs.
9. Incorporated a semi-automatic protein screening program.
10. Cross-referenced TCDB with HOGENOM (http://pbil.univ-lyon1.fr/databases/hogenom/acceuil.php), DIP (18), RefSeq (19), Entrez (20), Pfam (21), BioCyc (22), KEGG (23), PDB (24) and DrugBank.
11. Improved search tools that now separate results by system, cluster, family, superfamily and reference.
12. Implemented GBLAST, which provides a search tool designed to identify potential transporters in fully sequenced genomes or DNA segments (25–27).
13. Implemented Ancient Rep, which provides horizontal and vertical search approaches to find transmembrane repeat units within a single protein or a list of homologs, respectively (13).
14. Updated UniProtKB (28) cross-reference files with a continuously updated dynamic version as of 15 August 2013.
15. Provided links to DrugBank (29) allowing resolution to the well-known, validated human drug targets presented by Rask-Andersen *et al.* (30), as well as bacterial drug targets.
16. Implemented the Superfamily Tree programs, SFT1 and SFT2, which use tens of thousands of BLAST bit scores instead of multiple alignments, thus avoiding the pitfalls often encountered when determining the phylogeny of distantly related proteins (31–33). While SFT1 constructs trees allowing visualization of individual proteins, SFT2 allows depiction of family/subfamily relationships (31–33).
17. Provided a mechanism for user-generated input.

## GROWTH OF THE DATABASE (2010–13)

**Figure 2.**
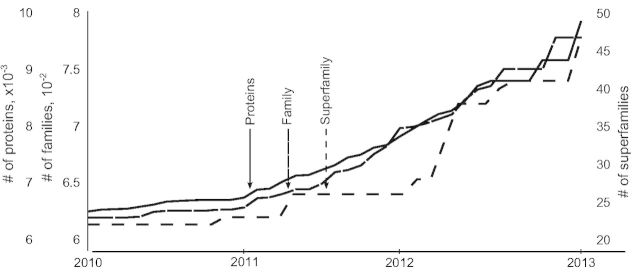
Growth of TCDB since August 2010. **(A)** Number of thousands of proteins (solid line); **(B)** number of hundreds of families (broken line); (**C)** number of superfamilies (dashed line). Numbers of proteins, families and superfamilies in TCDB as of 19 August 2013 were 9853, 778 and 49, respectively.

A file containing the current sequence set is available for download from: http://www.tcdb.org/public/tcdb. About 150 TC families are introduced each year, reflecting the extensive and continual manual curation work being conducted. Figure 2 shows the parallel growth of TCDB protein, family and superfamily compositions from 2010 to 2013. However, it should be noted that each year, several families in Class 9 are moved to classes 1–5 when sufficient information becomes available to allow definition of their mechanisms of action.

## SUPERFAMILY ADDITIONS (2011–13)

Analyses (34–43) have revealed distant relationships between preexisting TC families. These relationships have been integrated into TCDB as a hyperlink, and superfamily relationships are mentioned with hyperlinks in the description of each constituent family. The number of superfamilies that are either new or expanded (marked with superscript ‘a’ in Table 1) has more than doubled during the last 3 years (Figure 2), and the further expansion of such knowledge continues.

**Table 1.** Transport protein superfamilies in TCDB

1.	Aerolysin^a^
2.	Amino acid/Polyamine/organoCation (APC)^a^
3.	ATP-Binding Cassette-1 (ABC1)
4.	ATP-Binding Cassette-2 (ABC2), with the ECF sub-superfamily
5.	ATP-Binding Cassette-3 (ABC3)
6.	Bacterial bacteriocin (BB)^a^
7.	Bile/arsenite/riboflavin transporter (BART)^a^
8.	Cation diffusion facilitator (CDF)^a^
9.	Cation:Proton antiporter (CPA)
10.	Cecropin
11.	Circular bacterial bacteriocin (CBB)^a^
12.	Claudin^a^
13.	Corynebacterial PorA/PorH^a^
14.	Defensin
15.	Drug/metabolite transporter (DMT)
16.	Endomembrane protein translocon (EMPT)^a^
17.	Epithelial Na^+^ channel (ENaC/P2X)
18.	Gap junction (GJ)^a^
19.	General bacterial porin (GBP)
20.	Holin I^a^
21.	Holin II^a^
22.	Holin III^a^
23.	Holin IV^a^
24.	Holin V^a^
25.	Holin VI^a^
26.	Holin VII^a^
27.	Huwentoxin
28.	Ion transporter (IT)
29.	Lysine exporter (LysE)
30.	Major facilitator (MFS)^a^
31.	Major intrinsic protein (MIP)^a^
32.	Melittin
33.	Membrane attack complex/perforin (MACPF)^a^
34.	Mercury (Mer)
35.	Mitochondrial carrier (MC)
36.	Mycobacterial/nocardial porin (MspA)^a^
37.	Multidrug/oligosaccharidyl-lipid/polysaccharide (MOP) Flippase^a^
38.	P-type ATPase (P-ATPase)
39.	Phosphotransferase system, Asc/Gat (PTS-AG)
40.	Phosphotransferase system, Glc/Fru/Lac (PTS-GFL)
41.	Resistance-nodulation-cell division (RND)
42.	RTX-toxin
43.	T4 immunity (T4 IMM)^a^
44.	Transmembrane, inner membrane-17 (Tim17)
45.	Transporter/opsin/G protein-coupled receptor (TOG)
46.	TRC/TAMP-B (TRC/TAMP)^a^
47.	Outer membrane protein (OMP) insertase (YaeT/TpsB)
48.	Voltage-gated ion channel (42)
49.	Viral envelope glycoprotein (Env)^a^

^a^New or recently expanded superfamilies.

## ESTABLISHING HOMOLOGY BETWEEN PROTEINS USING TCDB-RELATED SOFTWARE

Affiliation with a family requires satisfying rigorous statistical criteria of homology. Superfamily status is based on the superfamily principle (44,45), stating that if protein A is homologous to protein B, and protein B is homologous to protein C, then protein A must be homologous to protein C, regardless of the degree of sequence similarity observed between proteins A and C. To avoid the concern of convergent evolution, the minimal length of aligned sequences to establish homology is 60 residues, and the comparison score must be at least 12 standard deviations using the GSAT program [see also Wakabayashi *et al.* (10)]. As the protein databases grow, this value must be increased (44–46). It should be noted that homology means ‘derived from a common evolutionary origin’. Homology is therefore an absolute term and does not require a specific degree of sequence similarity between any two protein sequences such as sequences A and C discussed above (45).

Summarizing, we have developed and perfected novel tools suited for the analysis of transporters (http://saier-144-21.ucsd.edu/). These are geared toward (i) superfamily recognition, (ii) detection of internal repeats, (iii) genome analyses of transporters (25,26,47,48), (iv) integral membrane topological analyses (31–33,49,50) and (v) family (38,51–58)/superfamily phylogenetic tree construction using two very different methods (31–33). These programs can be found in the ‘BioTools’ link of TCDB. A reference resource providing detailed information on these programs can be found in our Wiki (http://132.239.144.24) and in a chapter of a recent book edited by Christine A. Orengo (10).

## OTHER TRANSPORT DATABASES

Only TCDB is comprehensive, including transport systems from all living organisms, and only TCDB has been adopted by the IUBMB. However, several databases have been developed (see Table 2) which represent transporters in restricted groups of organisms, or are restricted to a certain category of transporter: (i) TransportDB (59) contains computerized annotations of transport proteins in organisms with fully sequenced genomes, and classifies them according to TCDB using a semi-automated pipeline. (ii) YTPdb (60) includes 298 *Saccharomyces cerevisiae* transporter proteins. It is organized by TC class, although TC#s are not provided. Each entry is a wiki where users can contribute. It is easy to use, but lacks the detailed text descriptions of sequences and families that can be found in TCDB. (iii) Aramemnon (61) provides manually curated protein descriptions for six plant species using a clustering algorithm that has been applied on a matrix of pairwise distances between sequences. (iv) The *Medicago trunculata* transporter database (62) focuses on transporters in a single plant genome based on TCDB. (v) ABCdb (63) contains lists of ABC transporters in prokaryotes in 21 families with functional predictions improved by the addition of references to TCDB. (vi) ABCISSE (64) tabulates 34 324 partners of 13 276 ABC transporter systems in 276 genomes. It is built around a phylogeny of 34 families of ABC ATPases (not the membrane constituents), organized in three classes with text descriptions only for the families. TCDB currently includes 92 families of ABC transporter systems, 35 families of uptake porters, 45 families of prokaryotic exporters and 12 families of eukaryotic exporters. (vii) The Human ATP-Binding Cassette Transporters (http://nutrigene.4t.com/humanabc.htm) categorizes 49 transport systems into subfamilies A–G (65). It is a list, not a database, providing some links to other resources. All these human transporters have been entered into TCDB. (viii) SLC tables (66) classify secondary carriers in mammals, especially human and mouse. SLC contains 52 families compared with 115 in the equivalent TC subclass of 2.A. We have interconnected the two systems and included all human carriers in TCDB. The tables revealing the family relationships between the TC and SLC systems can be found at the top of subclass 2.A in TCDB. The worm SLC database lists multiple homologs of individual SLCs in *Caenorhabditis elegans*. (ix) The membrane proteins of known three-dimensional structure database (67) contains 379 entries, that constitute a subset of PDB, not all of them transporters. PDB entries are grouped broadly by type. (x) The UCSF PMT is a SNP database, showing schematic diagrams of transporters with SNPs marked out in the sequence but does not attempt to provide TC numbers. (xi) The ARDB contains antibiotic resistance genes, providing a list of four types of multidrug resistance transporter types: ABC (TC# 3.A.1), MFS (TC# 2.A.1), RND (TC# 2.A.6) and SMR (TC# 2.A.7.1).

**Table 2.** List of known transporter databases

Name	URL	Interconnected with TCDB
TransportDB	http://www.membranetransport.org/	Yes
YTPdb	http://ytpdb.biopark-it.be	Yes
Aramemnon	http://aramemnon.botanik.uni-koeln.de/	No
*M. trunculata TDB*	http://bioinformatics.cau.edu.cn/MtTransporter/browse.php	Yes
ABCdb	https://www-abcdb.biotoul.fr/	Yes
ABCISSE	http://www1.pasteur.fr/recherche/unites/pmtg/abc/database.iphtml	No
Human ABC TDB	http://nutrigene.4t.com/humanabc.htm	Yes
SLC tables	http://www.bioparadigms.org/slc/intro.htm	Yes, in TCDB
Worm SLC db	http://wwwWormSLC.org	No
MP struc	http://blanco.biomol.uci.edu/mpstruc/	No
UCSF PMT	http://pharmacogenetics.ucsf.edu/	No
ARDB	http://ardb.cbcb.umd.edu/	No

## HARMONIZATION AND FUTURE GOALS

The most important goals we have identified for future development of TCDB include (i) the creation of an ontology for the TCDB database, (ii) improving our integration with Pfam and (iii) streamlining the use of phylogeny and synteny information to provide functional predictions. Some of the new functions will be implemented as links, and some as software. Synteny should probably be implemented as links, because the information is often already available elsewhere (Microbes Online, JGI’s intuitive resource IMG, SEED and RegPredict). Pfam may prove more difficult, because many families in Pfam are incomplete or not appropriately arranged in clans. Working with Pfam as we have in the past (69), we plan to improve upon the transport protein section of this database.

It is well-known that many families that include domain duplicated transporters do not accurately reflect the domain borders in the way hidden Markov models (HMMs) have been trained (68). Currently, we do not show ‘repeat units’ in TCDB, even though this information is presented in many of our publications. We will continue to work with Pfam to integrate and coordinate information in both databases in a systematic way (69). Ideally, such a process should be automated or semi-automated.

Another worthwhile goal is to establish the user base so we can serve the needs of the scientific community more effectively. We plan to collect more access statistics to understand the needs of the user community. Google Analytics was installed in 2011, but improvements are required so we can recognize which TCDB features are most used.

One million PubMed abstracts are created every year, and 10% of the 2012 abstracts were not indexed as of May 2013. Other databases that link to TCDB, such as EcoGene (70), manually review literature. ‘Transporter’ is a MESH term PubMed uses, but there is a 6-month delay to add MESH terms, and sometimes the word ‘Transporter’ is not obvious from the title. TCDB uses machine learning classifiers, as well as keyword searches which are continuously extracted from TCDB and used as search terms to identify relevant articles. We are considering new ways for users to provide sequence data and information either with or without the use of email; suggestions by email could be used as test sets to evaluate the efficiency of an automated process. We are also considering implementing links for reference, sequence and information input from users. Adding a feature allowing TCDB to be searched as a library of HMMs is also under consideration. Current TCDB users report that the present system of presenting search results is satisfactory, but we constantly strive to improve the database, and suggestions from users are most welcome.

TCDB needs an ontological hierarchical system and a controlled vocabulary. EBI’s ChemDB (71) has created a chemical classification system, and we have already set up a prototype which can be accessed from this link: http://www.tcdb.org/ontology/. The substrate text needs to be extracted from the description and then correlated with ChemDB. One system already exists, but due to inconsistencies in the description, it has been difficult to implement. If we could link with gene ontology, TC numbers would be more accessible. Another important area for improvement concerns user access to the most recent entries. Perhaps TCDB should have ‘recent releases’, such as those of Pfam. Since we already track protein histories, adding this feature would not be difficult. Some basic statistics, where database growth can be followed, are already available at: http://www.tcdb.org/search/index.php.

We are currently undertaking the development of standardized workflows to confirm homology results from TCDB’s in-house statistical methods, based on structural superimposition and HMM:HMM comparisons. For instance, we use structural superimposition in addition to sequence statistical analyses to identify or confirm structural and evolutionary relationships between members of a superfamily (40). This helps to establish reference points in structural space for homology detection.

## CONCLUSION

In 2006, TCDB contained ∼3000 proteins, classified into ∼400 families, but in 2013 it exceeded 10 000 proteins in ∼750 families. The availability of TCDB has allowed major basic research advances including answering fundamental biological questions, determining the routes of evolution taken for the appearance of these proteins, identifying superfamily relationships and allowing structural, functional and mechanistic predictions. Within practical limits, TCDB reflects the current state of our knowledge concerning its constituent parts.

## FUNDING

TCDB is supported by NIH [GM 077402-05 and GM 094610-01]. Funding for open access charge: NIH.

*Conflict of interest statement.* None declared.
